# Gender Effects on the Impact of Colorectal Cancer Risk Calculators on Screening Intentions: Experimental Study

**DOI:** 10.2196/37553

**Published:** 2023-06-12

**Authors:** Jungmin Lee, Mark Keil, Jong Seok Lee, Aaron Baird, Hyoung-Yong Choi

**Affiliations:** 1 J Mack Robinson College of Business Georgia State University Atlanta, GA United States; 2 Haslam College of Business The University of Tennessee Knoxville, TN United States; 3 HUFS Business School Hankuk University of Foreign Studies Seoul Republic of Korea

**Keywords:** colorectal, cancer, oncology, risk calculator, risk, perceived susceptibility, susceptibility, gender, intention, randomized, randomization, screening, perception, prevention

## Abstract

**Background:**

According to a 2020 study by the American Cancer Society, colorectal cancer (CRC) represents the third leading cause of cancer both in incidence and death in the United States. Nonetheless, CRC screening remains lower than that for other high-risk cancers such as breast and cervical cancer. Risk calculators are increasingly being used to promote cancer awareness and improve compliance with CRC screening tests. However, research concerning the effects of CRC risk calculators on the intention to undergo CRC screening has been limited. Moreover, some studies have found the impacts of CRC risk calculators to be inconsistent, reporting that receiving personalized assessments from such calculators lowers people’s risk perception.

**Objective:**

The objective of this study is to examine the effect of using CRC risk calculators on individuals’ intentions to undergo CRC screening. In addition, this study aims to examine the mechanisms through which using CRC risk calculators might influence individuals’ intentions to undergo CRC screening. Specifically, this study focuses on the role of perceived susceptibility to CRC as a potential mechanism mediating the effect of using CRC risk calculators. Finally, this study examines how the effect of using CRC risk calculators on individuals’ intentions to undergo CRC screening may vary by gender.

**Methods:**

We recruited a total of 128 participants through Amazon Mechanical Turk who live in the United States, have health insurance, and are in the age group of 45 to 85 years. All participants answered questions needed as input for the CRC risk calculator but were randomly assigned to treatment (CRC risk calculator results immediately received) and control (CRC risk calculator results made available after the experiment ended) groups. The participants in both groups answered a series of questions regarding demographics, perceived susceptibility to CRC, and their intention to get screened.

**Results:**

We found that using CRC risk calculators (ie, answering questions needed as input and receiving calculator results) has a positive effect on intentions to undergo CRC screening, but only for men. For women, using CRC risk calculators has a negative effect on their perceived susceptibility to CRC, which in turn reduces the intention to sign up for CRC screening. Additional simple slope and subgroup analyses confirm that the effect of perceived susceptibility on CRC screening intention is moderated by gender.

**Conclusions:**

This study shows that using CRC risk calculators can increase individuals’ intentions to undergo CRC screening, but only for men. For women, using CRC risk calculators can reduce their intentions to undergo CRC screening, as it reduces their perceived susceptibility to CRC. Given these mixed results, although CRC risk calculators can be a useful source of information on one’s CRC risk, patients should be discouraged from relying solely on them to inform decisions regarding CRC screening.

## Introduction

According to a 2020 study by the American Cancer Society, colorectal cancer (CRC) represents the third leading cause of cancer both in incidence and death in the United States [1]. CRC is similar to other types of cancer in the sense that the disease can be developing for some period of time without the patient knowing it. By the time a person has developed symptoms, the disease can be difficult to treat. Regular screening for early detection is therefore important. Nonetheless, screening in the case of CRC can be more effective than other types of cancers due to the slower progress from precancerous polyps to adenomas, the invasive cancerous polyps [2]. In fact, health research has accumulated much evidence that shows the effectiveness of CRC screening [3]. Unfortunately, CRC screening remains lower than that for other high-risk cancers such as breast and cervical cancer [1], and an estimated 37% of Americans who should have received CRC screening have not done so [2]. Although there are multiple options for CRC screening, colonoscopy has often been regarded as the gold standard among the options available and is often recommended to patients by their physicians [4].

Health risk calculators are one form of intervention used to encourage people to undergo cancer screening. Risk calculators for CRC are now readily available to anyone with a web browser. Although it has been suggested that “providing people with individualized risk estimates can encourage them to engage in health-promoting behaviors” [5], prior research suggests that risk calculators may not be that effective in increasing people’s intention to undergo CRC screening [3]. While some studies found that the use of health risk calculators increases individuals’ intentions to sign up for screening [6-9], other studies reported a negative or nonsignificant relationship between the use of risk calculators and intentions to sign up for screening [3,6,10]. In addition, some studies also measured perception of risk and reported that risk calculators actually lowered participants’ perceptions of risk [7,10,11].

Moreover, a meta-analysis by Portnoy et al [12] reveals that the use of a risk calculator is a strong predictor for the perceived susceptibility of health-related outcomes, and its effect size (*B*) is –0.65 (95% CI –1.13 to –0.16). These results suggest that, in general, using risk calculators decreases perceived risk of health-related issues. For example, Harle et al [10] found that, on average, individuals’ risk perceptions of prediabetes decreased by 2% after they received the results of individualized risk calculations [10]. Moreover, Losina et al [7] examined the efficacy of a personalized risk calculator on risk perceptions of knee osteoarthritis. They found that after using the calculator, participants’ perceived 10-year risk decreased by 12.9 percentage points to 12.5% and perceived lifetime risk decreased by 19.5 percentage points to 28.1%. In the context of colon cancer risk, research suggests that using risk calculators does not lead to expected benefits (ie, increasing risk perceptions). Specifically, Weinstein et al [11] found that correlations between actual and perceived risks of colon cancer were about the same between people who received personalized feedback and those who did not receive such feedback.

Given the inconclusive findings concerning the impact of risk calculators on intentions to sign up for CRC screening, further research is needed to probe this relationship and to shed light on the mechanism through which CRC risk calculators may influence individuals’ intentions.

One possible explanation for inconsistent research findings concerning the impacts of CRC risk calculators is that users may regard the output of such calculators, usually provided in percentage terms, to be so small that they perceive themselves as having a very low susceptibility to CRC. The lifetime risk of CRC in the general population is considered to be between 5% and 6% [13], and one study reported an average CRC risk calculator result for 10-year risk as 1.02% among a group of 509 patients undergoing colonoscopy [14]. Another possible explanation for the inconsistent research findings concerning CRC risk calculators is that some groups of people are likely to regard the risk as more concerning than other groups. Specifically, there is a substantial body of research indicating that women and men differ in their perceptions of risk [15]. Therefore, it is deemed important to consider gender and examine its role in understanding the impacts of CRC risk calculators.

Therefore, in this study, we examine the effect of using CRC risk calculators on individuals’ intentions to undergo CRC screening. In addition, this study aims to examine the mechanisms through which using CRC risk calculators might influence individuals’ intentions to undergo CRC screening. Specifically, this study focuses on the role of perceived susceptibility to CRC as a potential mechanism mediating the effect of using CRC risk calculators. As one of the constructs in the Theory of Planned Behavior, intention is defined as the effort one is willing to exert to reach a behavioral goal and is suggested as the “proximal antecedent to action” [16]. Perceived susceptibility is an important factor in shaping risk perceptions and is defined as an individual’s subjective probability that something, in this context CRC, will negatively affect him or her [17]. Finally, this study also examines how the effect of using CRC risk calculators on individuals’ intentions to undergo CRC screening may vary by gender. We note that we do not develop a priori hypotheses for two reasons: (1) prior findings regarding the effects of risk calculators have been inconsistent and (2) existing theory does not provide us with enough information to predict the moderating effect of gender with any precision (ie, whether it would be stronger for men or for women).

**Figure 1:**
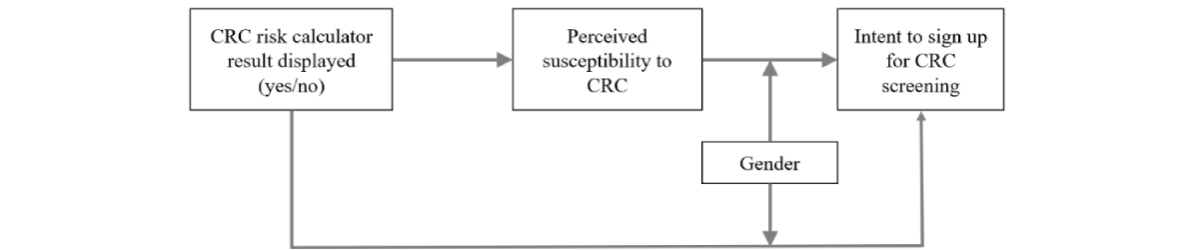
Research model. CRC: colorectal cancer.

In sum, we seek to address the following research questions: (1) how does providing an individualized risk score via a risk calculator influence an individual’s intention to undergo CRC screening and specifically what role does perceived susceptibility play? (2) Does gender affect the mechanism through which individuals respond to a personalized risk calculator score? Our research model is depicted in Figure 1.

## Methods

To address our research questions and test our research model, we conducted an experiment.

### Ethics Approval

For this study, we sought and received approval from the Georgia State University institutional review board (IRB; IRB number H19530, reference number 358302) at the university of the corresponding author where data were collected and managed. This study fell under the exempt study category based on the guidelines of the IRB. Before participants could participate in this study, they were asked to read the informed consent form that was approved by the IRB and indicate their willingness to participate by clicking the “Agree” button (the experiment was conducted in a web-based setting). The informed consent form explained the objectives of this study in layman’s terms without revealing any information about the experimental design. Specifically, participants were told that we are interested in studying the effect of personalized CRC risk on intention to sign up for CRC screening. We also explained in the informed consent form that participating in this study was completely voluntary. We did not collect any personal or identifiable data. In other words, the data were completely anonymous. In addition, the data were kept in password-protected computers. Finally, the participants received US $0.80 for their participation.

### Experimental Design

We used a pretest-posttest control group design with random assignment (see Table 1) in which both perceived susceptibility and intention to sign up for CRC screening were measured before and after the manipulation. Pretest measures were used to ensure that participants in treatment versus control groups did not differ in terms of perceived susceptibility and intention to sign up for CRC screening before participating in the experiment. Specifically, we conducted a *t* test comparing pretest measures of perceived susceptibility to CRC and intention to sign up for CRC screening between the 2 groups, and there was no statistically significant difference at the *P*<.05 significance level. Moreover, including a control group of individuals who did not learn their risk allowed us to create a tight experimental design in which the treatment and control group participants had exactly the same experience except receiving the risk score. Specifically, this allowed us to be confident that any differences found between the treatment and control group were due to having received a risk calculator score and not due to having gone through the act of answering the risk calculator input questions.

**Table 1:** Experimental design.

Participants	Assignment	Pretest	Manipulation	Posttest
Treatment group	R^a^	O_1_^b^	X^c^	O_2_
Control group	R	O_3_	N/A^d^	O_4_

^a^R: random assignment.

^b^O: observation.

^c^X: treatment received.

^d^N/A: not applicable.

Although both treatment and control groups provided inputs for the CRC risk calculator after the pretest, only the treatment group received the personalized CRC risk calculator result before the posttest. In contrast, participants in the control group received the risk calculator results at the end of the experiment (ie, after submitting their posttest responses). This constituted the manipulation. In other words, in the posttest, the control group reported their perceived susceptibility to CRC and their degree of intention to get screened for CRC right after the input process but before receiving the risk calculator result, whereas the treatment group was provided with the risk calculator output before responding to the perceived susceptibility and intention measurements. Both groups completed standard CRC risk calculator input questions. This design allowed us to examine whether receiving the CRC risk calculator results influences perceived susceptibility and intention to sign up for a colonoscopy.

### Recruitment

The experiment was conducted through the Qualtrics survey platform, and we recruited participants through Amazon Mechanical Turk (see Table 2). We restricted study participation to people aged 45 to 85 years, who live in the United States, and have health insurance (private health insurance, or Medicare or Medicaid). Health care practices and behavior vary by country and culture, and for our study, we wanted to focus on the United States. In addition, the financial burden is a major factor influencing CRC screening nonadherence [18,19]. Therefore, to address the possible confound of financial means, we included having health insurance as a requirement for participating in our experiment.

**Table 2:** Participants (N=128).

Participants	Men, n	Women, n	Total, n
Treatment group	20	42	62
Control group	26	40	66
Total group	46	82	128

Upon consent, participants were asked to answer questions about age, health insurance type, and whether they had ever had CRC screening. These 3 questions were used to filter out people who were younger than 45 years, who did not have any form of health insurance, and those who had already undergone CRC screening. Initially, a total of 219 Amazon Mechanical Turk users agreed to take part in the experiment, but 78 of them were filtered out by the initial screening question about age, insurance, and prior CRC screening experience. In addition, 13 participants failed to pass the attention check questions and thus were removed from the study. This resulted in a total of 128 usable responses for our analysis.

### Statistical Analysis: Power

A priori, the required sample size was calculated using G*Power (version 3.1.9.7) [20] assuming a medium effect size (*f*^2^=0.15), an α level of .05, and a power of 0.80, resulting in the required total sample size of 92 individuals. We based this on the meta-analysis by Portnoy et al [12], which found that the use of a risk calculator is a strong predictor for the perceived susceptibility of health-related outcomes, and its effect size (*B*) is –0.65 (95% CI –1.13 to –0.16). In other words, –0.65 is considered a medium effect size and we used this as a guideline. Our sample size of 128 exceeded this and was deemed to give us sufficient power.

### Risk Calculator

All participants were asked to provide the required inputs needed for a CRC risk calculator to assess their personalized risk for contracting CRC in their lifetime. To enable this process, we adapted the CRC risk calculator from the National Cancer Institute [21]. The calculator uses participants’ demographic, health, and lifestyle information including age, height, weight, dietary and physical activity, medical and family history to CRC, and cigarette usage for men and hormone usage for women. The calculator then provides a risk percentage expressing the lifetime chances of developing CRC.

The National Cancer Institute provides the SAS code for the risk calculator. We created the calculator using the code and integrated it with the web-based survey. One adaptation was made regarding the age group. The original calculator was designed for the age group of 50 to 85 years, but our calculator was modified to also include people aged 45 to 49 years. We made this modification based on the current CRC screening recommendations of the American Cancer Society [22]. Participants aged 45 to 49 years received the same outputs from the calculator that they would have received if they had entered the age of 50 years, as the SAS code on which our calculator was based did not yet reflect the updated screening guideline at the time we conducted the experiment.

### Measures

We posit in this study that intention to sign up for CRC screening is affected by one’s perceived susceptibility to CRC. The 2 constructs were measured before and after users provided inputs for the CRC risk calculator.

#### Intention to Sign Up for CRC Screening

Measures for intention to sign up for CRC screening were adapted from previous studies [22,23]. Participants were asked to respond to 5 measurement items, each on a 7-point Likert scale (1=strongly disagree; 7=strongly agree; see Tables S1 and S2 in Multimedia Appendix 1). Behavioral intentions are commonly used in health behavior literature as the primary dependent variable and are held to be predictive of actual behavior [24]. Cronbach α in this study was .96 for both the pretest and posttest intention to sign up for CRC screening.

#### Perceived Susceptibility to CRC

Measures for perceived susceptibility were also adapted from a previous study [25]. Participants were asked to respond to 3 measurement items, each on a 7-point Likert scale (1=strongly disagree; 7=strongly agree; see Tables S1 and S2 in Multimedia Appendix 1). Cronbach α in this study was .96 for pretest susceptibility and .98 for posttest susceptibility.

### Other Measures

After completing the posttest measures, participants were asked to respond to some additional questions involving demographics and control variables.

### Data Analyses

A comparison of the treatment and control group means was conducted using an independent samples *t* test in SPSS (version 25.0; IBM Corp). Statistical significance was defined as *P*<.05. Paired *t* tests were used for comparing pretest and posttest measures of perceived susceptibility to CRC and intention to sign up for CRC screening within each group. We used Hayes’ PROCESS macro for our main analysis to conduct a regression-based conditional process analysis of a moderated mediation model with 10,000 bootstrap samples [26].

## Results

### Descriptive Statistics

In our assessment of random assignment, we found no significant differences between the treatment and control groups in terms of mean age, objective CRC risk (CRC risk calculator score), or BMI at the *P*<.05 level. The descriptive statistics for the treatment and control groups and the *P* values for the mean comparisons are shown in Table 3.

**Table 3:** Descriptive statistics for the treatment and control groups (N=128; men 46 and women 82).

Variables	Control group (n=66; result not immediately received)			Treatment group (n=62; result immediately received)			*P* value
	Mean (SD)	Minimum	Maximum	Mean (SD)	Minimum	Maximum	
Age (years)	52.89(7.75)	45	74	53.73 (8.00)	45	69	.55
CRC^a^ risk calculator score	3.95 (1.41)	0.28	8.04	3.90 (1.57)	1.89	8.67	.86
BMI	28.33	18.29	59.76	28.41 (8.02)	19.63	67.31	.95

^a^CRC: colorectal cancer.

### Correlation Analysis

Next, we examined correlations among the key variables. We used posttest measures for the correlation analysis. As seen in Table 4, the intention to sign up for CRC screening was positively associated with perceived susceptibility to CRC (*r*=0.30, *P=*.001) and participants’ BMI (*r*=0.19, *P=*.04). However, it was not significantly correlated with any other variables, including age, gender, or whether the result from the risk calculator was received before the posttest measures. Age and gender did not show any significant correlation with other variables. Perceived susceptibility to CRC was negatively associated with whether the participant’s CRC risk score was received (*r*=−0.36, *P*<*.*001) but not with any other variables, suggesting that participants who received their personalized CRC risk score reported lower perceived susceptibility to CRC than those who did not. In addition, the correlation between pretest and posttest perceived susceptibility to CRC was 0.752 (*P*<.001). The correlation between pretest and posttest intention to sign up for CRC screening was 0.963 (*P*<.001; these are not shown in the table).

To determine whether participants’ perceived susceptibility to CRC and intention to get screened for CRC changed after the intervention, we examined the changes in perceived susceptibility and intention to sign up in each group using pretest and posttest measures. It was found that for each group the perceived susceptibility and intention to sign up for CRC screening decreased after the participants used the risk calculator but to a greater extent for the treatment group (see the post-pre difference in Table 5). These results indicate that receiving the risk calculator result (vs not receiving the result) can have differing effects on both the perceived susceptibility and intention to sign up for CRC screening.

In addition, the decreases in the perceived susceptibility and intention to sign up for CRC screening were statistically significant for the treatment group. In contrast, for the control group, the decrease in only the perceived susceptibility was statistically significant. A possible explanation for this pre versus post difference in perceived susceptibility, even in the control group, is that the input process associated with using the risk calculator can itself influence perceived susceptibility. As both groups responded to the input process, the act of going through a CRC risk calculator may have given them hints on the risk factors, and those with no family history or who live a relatively healthy lifestyle may have felt some relief even without receiving the risk output from the calculator.

**Table 4:** Correlation table.

Variables	Value, mean (SD)	Intention to sign up, *r* (*P* value)	Perceived susceptibility, *r* (*P* value)	Risk calculator results received (control: 0; treatment: 1), *r* (*P* value)	BMI, *r* (*P* value)
Intention to sign up for CRC^a^ screening	4.26 (1.66)	N/A^b^	N/A	N/A	N/A
Perceived susceptibility to CRC	2.71 (1.42)	0.30 (*.*001)	N/A	N/A	N/A
Risk calculator result received before posttest measures (control: 0; treatment: 1)	0.48 (0.50)	−0.02.81)	−0.36 (<*.*001)	N/A	N/A
BMI	28.37 (8.20)	0.19 (=.04)	0.06 (=.51)	0.01 (=.95)	N/A
Objective risk score	3.93 (1.48)	0.16 (=.07)	0.35 (<.001)	−0.02 (=.86)	0.35 (<.001)
Age	53.30 (7.85)	−0.08 (=.37)	0.17 (=.06)	0.05 (=.56)	0.06 (=.47)
Gender (men: 0; women: 1)	0.64 (0.48)	−0.05 (=.56)	−0.17 (=.06)	0.07 (=.40)	0.05 (=.55)

^a^CRC: colorectal cancer.

^b^N/A: Not applicable.

**Table 5:** Post-pre differences in perceived susceptibility and intention to sign up for CRC^a^ screening (N=128).^b^

Participants		Pretest, mean (SD)	Posttest, mean (SD)	Post-pre difference, mean (SD)	*P* value
**Treatment group (n=62)**					
	Perceived susceptibility to CRC	2.98 (1.16)	2.19 (1.26)	−0.79 (1.17)	<.001
	Intention to sign up for CRC screening	4.46 (1.66)	4.23 (1.71)	−0.23 (0.47)	<.001
**Control group (n=66)**					
	Perceived susceptibility to CRC	3.36 (1.26)	3.20 (1.39)	−0.16 (0.53)	.02
	Intention to sign up for CRC screening	4.36 (1.64)	4.30 (1.61)	−0.06 (0.41)	.21

^a^CRC: colorectal cancer.

^b^The results reported in this table are based on a set of paired samples *t* tests. As a robustness check, we also ran repeated measures ANOVAs and confirmed that the results from these analyses are consistent with the results obtained from the paired samples *t* tests.

### Main Analysis

A moderated mediation analysis using Hayes’ PROCESS macro (model 15, second-stage moderated mediation) was conducted to (1) test whether perceived susceptibility mediates the relationship between the treatment (of showing the risk calculator result or not) and intention to sign up for CRC screening and (2) examine the role of gender in moderating this relationship.

First, we examined the results from the analysis concerning the direct effect (Table 6). The results indicated that for men, receiving the CRC risk calculator result increased their intention to sign up for CRC screening (95% CI 0.23-1.87), but this was not the case for women (95% CI −0.77 to 0.70). These results suggest that gender moderates the direct effect of the treatment on intention to sign up for CRC screening. Second, we examined the results from the analysis concerning the indirect effect (Table 6). The results indicated that for women, receiving the CRC risk calculator result reduced their perceived susceptibility to CRC, which in turn reduced their intention to sign up for CRC screening (95% CI −0.91 to −0.21). This mediation effect was not significant among men (95% CI −0.47 to 0.18). These results suggest that gender moderates the indirect effect (via perceived susceptibility) of the treatment on intention to sign up for CRC screening. Finally, we examined both the index of moderated mediation and the results of pairwise contrasts between conditional indirect effects (the difference between indirect effects for men vs women). The index of moderated mediation was −0.43 and was statistically significant (95% CI −0.91 to −0.01). The difference between the 2 indirect effects was −0.53 and was statistically significant (95% CI −0.91 to −0.01). These results provide further evidence supporting both the moderating role of gender and the mediating role of perceived susceptibility.

**Table 6:** Direct effect and conditional indirect effects of the CRC^a^ risk calculator output.^b^

		Effect (SE)	LL^c^ BCCI^d^	UL^e^ BCCI
**Direct effects**				
	Men	1.05 (0.48)	0.23	1.87
	Women	−0.03 (0.37)	−0.77	0.70
**Indirect effects (via perceived susceptibility)**				
	Men	−0.10 (0.16)	−0.47	0.18
	Women	−0.53 (0.18)	−0.91	−0.21

^a^CRC: colorectal cancer.

^b^As a robustness check, we conducted the same analysis with the inclusion of the following covariates: age, BMI, one’s belief on their likelihood of getting CRC in their lifetime, and objective risk scores of participants provided by the risk calculator. The results from this analysis with covariates were fully consistent with those reported in this table.

^c^LL: lower level.

^d^BCCI: bias corrected confidence interval.

^e^UL: upper level.

### Simple Slope and Subgroup Analyses

**Figure 2:**
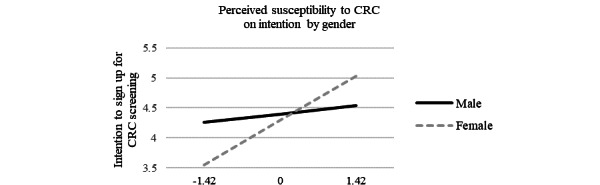
Gender difference on the relationship between perceived susceptibility and intention to undergo CRC screening. CRC: colorectal cancer.

**Figure 3:**
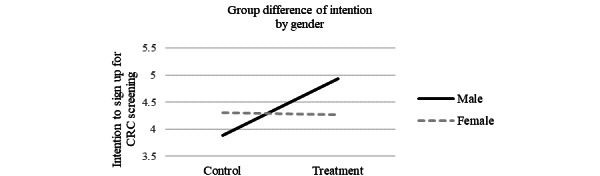
Gender difference on the effect of receiving the CRC risk calculator result on intention to undergo CRC screening. CRC: colorectal cancer.

We used simple slope tests to further examine the moderating role of gender on the relationship between perceived susceptibility and intention to undergo CRC screening (see Figures 2 and 3). Results indicated that perceived susceptibility to CRC had a significant positive effect on the intention to sign up for CRC screening for women (slope 0.53, SE 0.12; *P*<.001) but not for men (slope 0.10, SE 0.15; *P*=.51; Figure 2). These results provide additional insights suggesting that increased perceived susceptibility to CRC may lead to increased intention to sign up for CRC screening among women but not among men. The effect of receiving CRC risk calculator results on intention to sign up for CRC screening was also moderated by gender in that the effect was significant for men (slope 1.05, SE 0.42; *P=*.01) but not for women (slope −0.03, SE 0.37; *P=*.93; Figure 3).

**Figure 4:**
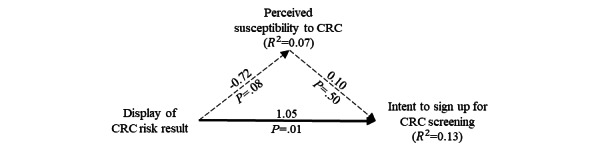
Subgroup analysis among men (n=46). CRC: colorectal cancer.

**Figure 5:**
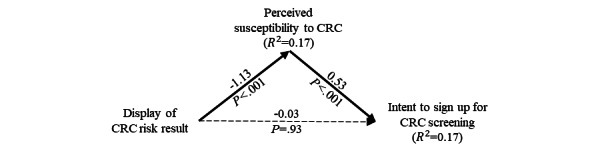
Subgroup analysis among women (n=82). CRC: colorectal cancer.

Moreover, results of subgroup analyses (shown in Figures 4 and 5) obtained by using the PROCESS macro (model 4) clearly show that the mechanism through which the risk calculator results influence intention to undergo CRC screening differs for men and women.

## Discussion

### Principal Findings

CRC screening is increasingly important, especially as CRC risk becomes greater for younger population segments, but compliance is suboptimal [27]. CRC risk calculators have the potential to promote CRC screening as they provide individualized risk scores that may positively impact intentions to undergo CRC screening. However, prior empirical research found that providing personalized risk feedback via risk calculators decreases perceived susceptibility to CRC [11]. Such results indicate that risk calculators may not be as useful for driving up compliance with CRC screening guidelines as one might think. In this paper, we investigated whether a CRC risk calculator can influence intention to undergo CRC screening by affecting an individual’s perceived susceptibility of contracting CRC. We further probed whether gender moderates the relationship between perceived susceptibility and CRC screening intention.

We found that among women, the effect of receiving CRC risk calculator results on their intention to undergo CRC screening is mediated by perceived susceptibility. Among men, the direct effect of receiving CRC risk calculator results was significant, whereas the mediating effect of perceived susceptibility was not. Although previous research studied the impact of using a risk calculator and receiving its results on perceived susceptibility (risk perceptions) [10,11] or the effect of perceived susceptibility on intention [28,29], little was known about the mechanism through which receiving CRC risk calculator results affects an individual’s intention to undergo CRC screening. Conditioning on gender, we show that among women, this relationship is mediated by perceived susceptibility and that among men, only the direct effect of receiving the CRC risk calculator result on their intention to sign up for screening was significant. Importantly, we also find that receiving CRC risk calculator results actually decreases CRC screening intention for women and that this is mediated through perceived susceptibility.

One interesting contribution of our study is the finding that perceived susceptibility may be central in explaining why the use of CRC risk calculators may not lead to a desired behavioral outcome. Furthermore, the finding that the risk calculator results influence men and women through different pathways and in different directions sheds light on why prior research has obtained inconsistent findings [6-11]. Using a direct and second-stage moderated mediation model, we found that gender moderates the mediating role of perceived susceptibility, such that the relationship between perceived susceptibility and intention to undergo CRC screening was significant only for women. We found that gender also moderates the direct effect of receiving risk calculator results, such that the direct effect was significant only for men. This indicates that gender differences should be considered when promoting CRC screening, suggesting that additional research is needed on how to successfully motivate CRC screening, conditional on gender.

### Implication for Practice

Our primary implication for practitioners is to be cautious when implementing CRC risk calculators as a primary intervention for promoting CRC screening, as the results may not be desirable. Although the individualized CRC risk scores may seem like useful information, relatively low CRC risk scores [13,14] likely cause users to perceive that their risk of contracting CRC is low, and this may cause them to forgo screening. We suggest that, at a minimum, risk calculator results should be paired with thoughtful communication from health care providers about the implications of the results and the importance of undergoing CRC screening.

We secondarily note that these findings have interesting implications for providing predictive model scores to individual health care consumers. In this study, we found that the effects of receiving such scores can vary by gender. The results indicated that for men, receiving the CRC risk calculator result increased their intention to sign up for CRC screening, but this was not the case for women. Our results suggest that health care providers may need to consider gender differences when discussing CRC risk calculator results with patients.

Finally, given the easy access that patients have to calculators that are available on the web, providers should educate patients so that the results provided by these calculators do not deter patients from receiving the recommended screening. Although risk calculators can be a useful source of information on one’s CRC risk, patients should be discouraged from relying solely on them to inform decisions regarding CRC screening.

### Limitation and Future Research

Although we have identified a mechanism that further explains how CRC risk calculator results affect intention to undergo CRC screening, our study has limitations. First, our study measures intentions rather than behaviors. Further work is needed to verify that our findings translate to actual behaviors. Second, although we found that men and women react differently to CRC risk calculator results, further work is needed to understand more deeply why this gender difference occurs. Third, there may be other moderators that were not included in our study that could be important. Future research could include additional constructs such as masculinity, fatalism, and anxiety to probe their effect in the context of CRC screening.

Finally, although our overall sample size (N=128) was larger than the calculated required sample size (n=122) at a medium effect size (*f*^2^=0.15), our sample exhibited gender imbalance, with almost twice the number of women (n=86) as men (n=46). Therefore, one avenue for future research would be to replicate our study with a larger and more balanced sample.

### Conclusions

Health risk calculators have the potential to promote healthy behavior by influencing participants’ risk perception. Through this study, we found that among women, perceived susceptibility to CRC mediates the relationship between receiving CRC risk calculator results and the intention to undergo CRC screening. Among men, the direct effect of receiving CRC risk calculator results was significant, whereas the mediating effect of perceived susceptibility was not. We also showed that the direction of the overall effect of receiving the CRC risk calculator result is positive for men and negative for women. In addition, as receiving the CRC risk calculator output was found to reduce perceived susceptibility to CRC, careful consideration on how to communicate such results is needed. Our findings suggest that interventions that influence perceived susceptibility may have unintended consequences on promoting CRC screening, underscoring the importance of communicating the result. Although the use of an individualized risk assessment tool can be a good addition to one-on-one communication with a health care provider, the messaging provided by both the tool and the clinician may need to be tailored to account for gender differences.

## Appendix

Multimedia Appendix 1 Measures for intention to undergo CRC screening and perceived susceptibility to CRC.

## Abbreviations

CRC: colorectal cancer
IRB: institutional review board

## Data Availability

The data set has been uploaded to a publicly available repository (Figshare). Our data can be found on Figshare [30].
