# Supplementary material for: Gender Effects on the Impact of Colorectal Cancer Risk Calculators on Screening Intentions: Experimental Study
Source: JMIR Form Res. 2023 Jun 12;7:e37553. doi: 10.2196/37553 (PMC10361457; doi:10.2196/37553)
Supplement: Multimedia Appendix 1 [file formative_v7i1e37553_app1.docx]

**Table S1.** Measures for intention to undergo CRC screening

| **Behavioral Intention** | I intend to sign-up for CRC screening if offered. | Adapted from Sheeran et al., 2001  (Cronbach  α =.93) | 7-point  Likert-type scale  (1=strongly disagree; 7=strongly agree) |
| --- | --- | --- | --- |
|  | If I had the opportunity, I would sign-up for CRC screening. |  |  |
|  | If I was offered a CRC screening, I would try to sign-up. |  |  |
|  | I intend to sign-up for CRC screening. | Adapted from Venkatesh and Davis, 2000 (Cronbach  α =.87) |  |
|  | I predict that I will sign-up for CRC screening in the short term. |  |  |

**Table S2.** Measures for perceived susceptibility to CRC.

| **Perceived Susceptibility** | When it comes to the likelihood of getting CRC, I believe that: | | |
| --- | --- | --- | --- |
|  | My risk of getting CRC is high. | Adapted from  Chen and Zahedi, 2016  (Cronbach α=.86) | 7-point  Likert-type scale (1=strongly disagree; 7=strongly agree) |
|  | The likelihood that I would get CRC is high. |  |  |
